# On Affections of the Mucous Membrane of the Digestive Organs

**Published:** 1821-06-01

**Authors:** 


### Medico-Chirurgical Review,

AND

JOURNAL OF MEDICAL SCIENCE.

(Analytical Series.)

Nec tibi quid liceat sed quid fecisse decebit

Occurrat mentemque domat respectus honesti. CLAUD.

VOL. II.] JUNE 1, 1821. [No. 5.

### I.

### AFFECTIONS

OF THE

### MUCOUS MEMBRANE OF THE DIGESTIVE ORGANS.

1. *Histoire des Phlegmasies, ou Inflammations Chroniques. Vol.* II. *De L'inflammation de la Membrane Muqueuse des Vois Digestives.* Par F. J. V. BROUSSAIS.

2. *Researches on the Pathology of the Intestinal Canal. On the Diseases of the Mucous Membranes.* By JOHN ABERCROMBIE, M.D. [*Ed. Journal,* 84.]

3. *Sur la Structure de la Membrane Muqueuse des Intestins dans l'Homme et dans quelques Animaux.* Par M. MEKEL, Professeur à l' Université de Halle. [*Journal Complimentaire, Septembre* 1820.]

4. *Sur les Phlegmasies Folliculaires.* Par le Docteur AIME GRIMAUD. [*Journal Complimentaire, Decembre* 1820.]

"Inflammatio quidem, ubicunque sit, non sine periculo habenda; periculosior tamen, ubi membranaceas occupaverit partes, ob exquisitum, quo pollent, sensum ac consensum gravissima et funesta simul symptomata adsociantur." *Hoffmani Opera, Tom.* III. *p.* 223.

IN the second number of this Series, we took up the subject of the *serous,* or peritoneal coverings of the intestines and abdominal organs generally—in the third number, the *mucous* membrane of the lungs—and in each, we endeavoured to convey, as far as possible, to our brethren in this country, the opinions and observations of M. Broussais, one of the most celebrated pathologists on the Continent. It is, however, on affections of the mucous membrane of the *digestive organs* that M. Broussais has most distinguished himself; and this consideration, together with the great importance of the subject, will lead us into a pretty extensive analysis of the second volume of this illustrious foreigner, while we shall, at the same time, endeavour to collect and concentrate as much valuable information as possible, from other authentic sources, on the portion of pathology now under investigation.*

M. Broussais justly observes that, when we consider the number and variety of extraneous substances, more or less stimulant, which are constantly traversing the mucous membrane of the digestive organs, it is really wonderful that we have not more frequently the phenomena of inflammation produced there. But, if we reflect on the exquisite sensibility of many portions of this membrane, and the numerous sympathies which subsist between it and almost every other part of the frame, we shall be convinced that, although actual inflammation is less frequent here than in the mucous membrane of the lungs, yet, that the morbid phenomena resulting from *irritation* in the primæ viæ, are infinitely more numerous and distressing than all the rest of the catalogue of human diseases collectively. We have, says M. Broussais, continually before our eyes, whole crowds of people who spend their time in tormenting the stomach with every thing burning and irritating which the animal and vegetable kingdoms can produce; and our books of pathology are filled with discussions of gastric and bilious derangements. If a drunkard dies of inanition, from defect of digestion, we are told of the loss of tone, or induration of the fibres of the stomach:—If he becomes dropsical—or dysenteric—the same explanation. Yet if we examine the symptoms exhibited by this class of patients, we shall find them correspond

---

* The critical expositions of M. Broussais' doctrines which have been drawn up in France, and re-published in England, convey little or no idea of the valuable facts and observations on which those doctrines are founded. They are shadowy insubstantial outlines magnified by generalities, but devoid of all useful and tangible particulars. Like criticisms in general, they have separated the grain from the chaff—leaving the grain behind. On coming to the examination of M. Broussais' work after perusing these *exposés* we found ourselves utter strangers to the real nature and true value of the publication. We are much mistaken if most of our readers are not in the same predicament.

exactly with what Pinel, in his *Nosographie Philosophique*, has laid down as characterizing chronic inflammation of the stomach and bowels.

Of the four phenomena exhibited in the living body, during inflammation, two only can leave traces in the dead—these are, *redness* and *swelling*—ulceration being a consequence of these. Whenever M. Broussais discovered these traces in the mucous membrane of the stomach, *post mortem*, he made diligent enquiries whether the other two phenomena, *heat* and *pain*, had been present during life. In a great majority of instances this was found to be the case; but when it was doubtful, Broussais examined very strictly, other patients similarly affected with those who died, and he constantly observed proofs of a morbid increase of sensibility in those tissues which, on dissection, shewed redness and tumefaction. Here then were three of the phenomena of inflammation unequivocal; and as to the fourth—*heat*—it is not easy to ascertain its presence in *chronic* inflammations. It was, however, evidently developed when any irritating substances were taken internally.

Thus then, M. Broussais adds, the signs of phlogosis of the internal surface of the stomach are, 1^mo^ during life, certain lesions of function referrible to a surplus or morbid sensibility of the mucous membrane—2^ndo^ On dissection, redness and ulceration of this tissue.

M. Broussais is well aware, indeed, that many physicians will refuse the name of inflammation to the abovementioned redness of the membrane—unless it is arrived at a high degree of intensity, and accompanied by pyrexia. To these objections he replies by an ample presentation of facts, in which the links that unite the most evident with the most obscure gastric phlegmasiæ may, he thinks, be readily recognized. Our author then details the histories and dissections of twenty-seven cases, occupying between sixty and seventy pages of the second volume, before he enters on the regular description, etiology, pathology, and treatment of the disease. We shall follow an inverse order;—first presenting an analysis of the didactic and descriptive part, and then selecting a few examples in elucidation of principles embraced or assumed.

I. *Etiology*. A phlogosed state of the mucous membrane of the digestive organs in general, is produced by whatever throws an undue degree of excitement on that tissue—principally by the impression of the atmosphere on the external, and aliments on the internal, surface of the body. Previous disease also may sometimes excite this affection. The etiology differs somewhat according to the gastric or intestinal seat of the disease. Thus our author conceives that the *predisposing* causes of gastric phlogoses are such things as tend to accumulate the susceptibility or excitability of the tissue, whether by a *general* action on the system, or a *local* one on the organ itself. The *former* is exemplified by atmospheric heat—the *latter*, by the introduction of alimentary substances which have the property of keeping a greater degree of action or excitement in the stomach than is consistent with the general harmony of the system.

In respect to those predisposing causes which act on the system generally, M. Broussais makes several important observations on the heat and electricity of the atmosphere. The *former* of these agents renders more excitable the living fibre, and under high temperature reaction is more energetic than in low. When high temperature, however, is long-continued, or very great in degree, the excitability loses in power by exhaustion. Hence, old residents in tropical and other hot climates have a more languid circulation, and less reaction, in acute diseases, than the inhabitants of hyperborean latitudes. Our author believes that, although the stimulus of atmospheric heat on the skin causes an increased secretion from that extensive surface, and consequently draws an over-proportion of fluids towards the periphery, yet that the irritability of the mucous membrane of the primæ viæ is, at the same time, increased, and rendered more liable to take on inflammatory action, when stimulants or irritants are improperly applied. M. Broussais admits that, under these circumstances, there is a corresponding increase of the biliary secretion—but considers it as the *effect*, and not the cause of irritation in the mucous membrane—"et la surabondance de la secretion bilieuse, autre *effet* necessaire de l'irritation de la muqueuse." We look on the phenomenon in quite another light—we consider the increased secretion of bile as the effect of increased stimulation on the surface—and the irritation in the primæ viæ as often the effect of this redundant and vitiated biliary secretion. We are glad to find the *fact* (however explained) confirmed by the testimony of a Broussais, who is equivalent to a score of closet sceptics among our fire-side travellers and practitioners of the present day.* Our author's

* Our intelligent readers need not be told that all extremes approximate, and that intense cold, and more especially alternations of cold and heat, by driving the blood occasionally from the surface to the interior organs, produce the same effect as intense heat, in disposing to visceral inflammation.

reasonings on the action of electricity in predisposing the mucous membranes to phlogosis, are not very clear to us, and therefore we shall not bewilder ourselves or our readers by noticing them.

The predisposing causes which act *directly* on the stomach and bowels, are, as we before said, the stimulating substances swallowed by the mouth, for whatever purposes. These may produce phlogosis of the mucous membrane, even without the aid of predisposition from atmospheric causes acting on the surface. The following passage may be perused with advantage by more than our colonial readers.

"If men would take care to diminish the stimulants which they swallow, in proportion to the increased excitability of the stomach produced by the atmospheric heat of our summers, or of tropical climates, until they became *acclimatés*, they would assuredly escape inflammatory affections. But when they sit down to table, they forget the precepts of Hygiene. They will retrench nothing from their former habits of indulgence. They swallow the same quantities of meat, spices, wine, coffee, liqueurs, as though they lived in the frozen zone, or their stomachs were in an ordinary state of excitability. Nay, such is the force of prejudice, that they consider this regimen as necessary to resist the debilitating effects of the climate! If, after an incendiary repast of this kind, a devouring thirst is kindled up, they would, if possible, quench it with more wine and spirits; but happily Nature compels them to have recourse to simpler fluids—and thus the antidote is every day opposed to the poison." Tom. II. *page* 191.

Our author goes on to enumerate the long list of "dishes tortured from their native taste," and inebriating liquors which especially among the predisposed, lead, sooner or later, to inflammatory affections of the digestive apparatus. In this list he very properly includes also the farrago of stomachic tinctures, and quack medicines which the credulous and unwary are constantly pouring down their throats, for the *purpose* of relieving, but actually with the *effect* of aggravating the states of irritation prevailing in the chylopoietic organs. Among the predisposing causes also may be classed unpleasant emotions of the mind, especially grief.

It is almost needless to say, that these *predisposing* causes, if long continued, or excessive in degree, become *exciting* causes of the inflammatory state. What has been said of the predisposing and exciting causes of gastritis will apply, as nearly as possible, to enteritis. M. Broussais makes many acute observations on the effects of atmospheric impressions, and the reception of miasmal impregnations in the system, in the production of diarrhœal and systematic affections; but his remarks are familiar to the English, who have long ago investigated these points. It may be proper to state, that M. Broussais considers dysentery as neither more nor less than inflammation of the mucous membrane of the intestines—and he makes an increased secretion of bile an occasional cause of the disease—an assertion that convinces us he never laboured under dysentery himself, or examined with care the stools of many dysenteric patients. Till within these few years an increased secretion of bile was considered the sole *cause* of cholera; but now it is pretty well proved, that it is a mere *effect*, and sometimes not at all present in the disease. In his etiology of irritation and phlogosis of the gastro-intestinal lining, M. Broussais includes almost every thing which we place in the etiology of fever, as the effluvia from crowded bodies of men, unhealthy food, depressing passions, great fatigue, poverty, &c. &c. This must be carefully borne in mind; for the reader is to recollect, that we are not exclusively on the subject of common unequivocal gastritis, especially as effecting the *peritoneal* covering of the organ, but on those irritated or phlogosed states of the *inner* surface of the digestive tube, accompanying (whether as cause or effect) a great portion of what are termed idiopathic fevers. The reader may not go the whole length of M. Broussais' doctrines; but it will be highly to his advantage to study with the profoundest attention, and divested of prejudice, the contents of this analysis. We shall now proceed to the development of symptoms in these phlogoses of the mucous membrane of the digestive tube.

II. *Symptomatology of Gastritis.* The first premonitory symptom is generally a sense of heat in the region of the stomach, during gastric digestion—at first, of a rather agreeable kind, ceasing when the stomach is empty, and succeeded by a farther craving for food. After these prelusive phenomena have continued for some weeks, or even months, according to the intensity of the causes, the person begins to perceive that this sense of gastric heat becomes unpleasant, and is coupled with a sympathetic heat of the skin, which is dry and rough. The tongue becomes dry and hotter than natural, with slight sore throat, broken rest, nervous irritability, heat and pain in the head. Now commences a kind of aversion to animal food and fermented liquors—sometimes thirst. When the disease sets in unequivocally, after these premonitions, it exhibits two forms, determined, as it would seem, by temperament.

A. *Acute Gastritis of the Mucous Membrane.* The first symptom is sometimes a violent vomiting, resembling cholera morbus. The patient throws up every thing that he swallows; then bilious, mucous, or even sanguineous looking matters; going very frequently to stool at the same time. Fever is a necessary accompaniment of this form of the disease. Sometimes gastritis declares itself without vomiting—but always with violent pyrexia, not preceded, in Broussais' experience, by a cold stage or shivering. The patient complains of a burning internal heat, and generally of a soreness in the pharynx. The tongue appears red and clean, or covered with mucus, except when the patient has been some time without drink. The thirst is considerable—the desire for cold acidulated drink is as great as the aversion for every other kind of liquid. If the phlogosis does not extend to the intestinal tube, there is constipation.—If it reach the colon, there is diarrhœa with tenesmus. There is deep-seated pain in the epigastric, and especially in the right hypochondriac region, but not exasperated without a certain degree of pressure. This pain is sometimes lancinating, and accompanied by a sense of constriction. It manifestly diminishes after the patient has swallowed cold aqueous drink, especially if acidulated. Very often the vomiting ceases, in a few days, although the other symptoms persist. At other times it continues or supervenes, in the course of the disease, and the patient is harrassed with constant nausea, which appears to him to be occasioned by some globular body rising upwards, and painfully compressing the lower part of the chest. Each fit of vomiting is followed by a temporary ease, of very short duration, the patient incessantly demanding emetics—a symptom still more common in peritoneal inflammation than in acute gastritis. The absolute impossibility, which the patient supposes, of swallowing any thing, appears referrible to the contracted and highly irritable state of the upper orifice of the stomach. Such are the principal symptoms in acute gastritis; but several of them may be absent—even pain itself does not exist, in some cases, where the inflammation is most intense. Our diagnosis must therefore be assisted by a rigid observance of the *sympathetic* troubles produced by this phlogosed state of the mucous membranes of the digestive organs. The *first* class of these appertains to the head, affecting the functions of the senses, and the movements of the voluntary muscles. Head-ache may or may not exist. Aberrations of the intellect, corresponding with the moments of greatest suffering, are more steady in their appearance.

"I have seen," says our author, "men as completely delirious as in fevers the most malignant, or phrenitis itself."

In such cases too, we often see the conjunctiva red, the eye inflamed, and the features altered. In proportion as the disease advances, and the sufferings increase, the attention becomes estranged, till coma ensues. In the mean time we observe irregular contractions of the facial muscles, grinding of the teeth, subsultus tendinum, and various convulsive movements. The patients throw off the bed-clothes, when they are sensible, complaining that the internal heat which devours them is ten times more insupportable when the chest is covered. They try all kinds of positions in bed; sigh deeply; and shew in their countenances the expression of intense agony. If they are questioned respecting the nature and seat of their pains, they apply the hand to the epigastric region, but cannot clearly describe their sufferings—the sense of internal burning is the only one which is distinct to them. We must therefore ground our diagnosis on the tout ensemble of the symptoms, and especially on the instantaneous relief produced by cooling drink. The muscular force is not *exhausted* in these cases, for the strength is quickly recruited after a crisis—which is not the case in those malignant and typhous fevers resulting from deleterious miasms.

In respect to the *respiratory system*, we observe sometimes a cough, with teazing pain; a glairy or mucous expectoration streaked with blood, or white, like that of peripneumony, at the period of resolution; a general pain in the chest; a laborious respiration in sanguineous subjects. The voice is often lost from a sympathetic paralysis of the laryngeal muscles.

During the first days of acute gastritis the pulse is full, hard, and often as strong as in pneumonia, particularly if the pectoral symptoms abovementioned are present—a proof of sanguineous plethora in the pulmonary parenchyma. In lighter shades of gastritis, and when the vital powers have been reduced by pain, the pulse is sharp, irregular, or even intermittent—towards the close of life imperceptible. Heat of skin is considerable, during the violence of the acute stage. M. Broussais has always found it dry and harsh. The skin is cold when the disease is on the decline—and cannot be brought to a natural warmth when the disease is verging to a chronic state. The cutaneous secretions are suppressed; and the breath is fetid in a few days after the circulation becomes much increased.

B. *Chronic Gastritis.* This may be a primitive affection, or the sequela of an acute attack. It is produced by the same causes as acute gastritis; but from peculiarity of constitution, or force of cause, it is unaccompanied by those violent commotions in the system which arrest the attention in the other species.

"The patient complains of pain across the base of the chest, deep-seated in the epigrastric and hypochondriac regions—generally more considerable in the right side, and sometimes so high up as to be thought in the chest. This pain is constant and very troublesome —sometimes burning, lancinating, pricking, and confined to a very circumscribed spot, especially when the stomach contains any acrid or irritating substances. It is very frequently accompanied by a sense of constriction. Some patients complain of feeling as though a ball of large size were pressing against the diaphragm—others as if a bar were fixed across the stomach, preventing their swallowing food or drink. Of all these sensations the lancinating and stinging pains are those which acquire the greatest degree of intensity. The others are so faint that the patient seldom demands relief from them till the strength becomes considerably reduced." 214.

The appetite always fails, and when the disease exists in its greatest degree, there is a general abhorrence of food. When there is any remains of appetite, the digestion is quite imperfect. Aliments are usually thrown up soon after eating—especially if too much food, or food of a stimulating nature have been swallowed. Those who, from a milder degree of the disease, or idiosyncrasy of stomach, do not vomit, are oppressed, during the gastric digestion, with a sense of load at the stomach, nausea, acid, corrosive or fetid eructations, rumination, and exasperation of the usual pain. There are some patients who only experience eructations, inquietude, malaise, and mental perturbation. The pulse rises a little, and the skin warms, during gastric digestion, but sink to their usual level when the digestive process is finished. For a considerable time the bowels are as costive, as though a scirrhus of the pylorus existed; but ultimately, in the majority of cases, there is diarrhœa, with colic, tenesmus, and stools mixed with blood—a proof of the extension of the disease. Then the breath and even the perspiration exhale an odour manifestly steroraceous.

These sufferings, even when not very severe, are badly borne by the sick, who become dejected, impatient, taciturn, discontented, and not disposed to enter into the details of their ailings. They have an air of suffering in their countenance; the conjunctiva, lips, and cheeks, being of a deep red colour, verging towards that of tincture of logwood, as are also the tongue and whole interior of the mouth, excepting along the centre of the former, where a thin mucous list may be seen. In a few subjects M. Broussais has observed, the tongue very much loaded, the breath offensive, and a bitter taste in the mouth—but these are exceptions, and the diagnosis must be drawn from the *tout-ensemble* of the symptoms, not from any one class exclusively.

As soon as chronic gastritis is completely established, the cellular and adipose membrane becomes nearly absorbed, with but little diminution of the muscles; when these last are much extenuated, the disease is without hope. At all times, however, the skin is drawn tight over the muscles, sinking in in their interstices, so that it cannot be pinched up but with difficulty, even in those places where it is usually very relaxed. In no other species of marasmus has M. Broussais seen this degree of adhesion so strongly marked. This character of the skin, together with its colour, being a brown, inclining to yellow, offer two of the most constant diagnostic signs of chronic gastritis.

The pulmonary system suffers very little in this species of the disease, with the exception of a slight stomach-cough occasionally. Nor is the circulation so much influenced, at the beginning, as to evince any appreciable febrile movement. When the disease has made progress, then the pulse becomes hard and frequent; the skin, at the same time, being hot, and dry to the touch. There is always an evening exacerbation, with agitation and restlessness. If this train continues unchecked, prostration of strength soon ensues, and the gastritis, in fact, passes into the acute form. If, however, the febrile movement is only marked by frequency of pulse, without heat of skin—or if the patient only experiences a few hours of heat towards the evening, or during digestion, the malady may continue chronic. In all cases, if long protracted, the febrile symptoms subside—and the evening exacerbation ceases to be sensible. Then the skin becomes cold, and of the colour before described, with perceptible wasting of the body. When diarrhœa is added, the cessation of pyrexial phenomena is still more sudden and complete.

III. *Phlogosis of the Mucous Membrane of the Intestines, or Dysentery.* M. Broussais observes that, we rarely find, in the bodies of those patients who have died with diarrhœa, any signs of phlogosis of the internal surface of the small intestines. Such inflammation is usually found in connexion with gastritis—indeed, where the mucous membrane of the small intestines is found inflamed at all, there is generally inflammation throughout the whole canal, from the cardiac orifice of the stomach to the anus. M. B. in this section, speaks only of inflammation as affecting the colon, which he divides into acute and chronic, though he seems to feel, that the shades so blend into one another occasionally, as not to be distinguished but in well marked cases.

A. *Acute Species.* In this case there are few premonitory symptoms, especially if the disease be very acute. In its highest degree, it is, according to M. Broussais, DYSENTERY. In our author's symptomatology, there are suspicious passages—as, for instance, his mentioning the discharge, at first, of *fœcal* stools, and afterwards, of *bilious* ones. Such occurrences we have not observed in one case in fifty, and we have seen more than two thousand cases of dysentery. We can allow, however, for these inaccuracies—or perhaps M. Broussais' patients were differently situated from those of our countrymen here, or in the colonies.

The phlogosis of the colon, M. Broussais has seen terminate, in gangrene, in the course of a few days, without any other mark of fever than a certain precipitation in the pulse, without any heat of skin. If the patient be vigorous, plethoric, or irritable, the febrile heat is strongly developed, after rigors more or less apparent from the beginning. Then the dysentery is acute and febrile, like the acute gastritis before described. The symptoms of acute dysentery need not be detailed—nor does our author enter into the consideration of complications of this disease with continued fever.

B. *Chronic Colonitis.* 1^mo^ Chronic *secondary* diarrhœas are not uncommon symptoms, sometimes a sequel of acute dysentery, and too often, in such cases, the consequence of mal-treatment. Most of these secondary bowel complaints have, at one period or other, symptoms in common with dysentery, as bloody stools, tenesmus, and some febrile movements in the system. 2^ndo^ *Primitive* chronic diarrhœa. A man, apparently well, becomes affected with bowel complaint, which sets in without fever or pain, and lasts a longer or shorter time, exhausting his strength and flesh, yet without causing any considerable disorder in the harmony of the other functions. This diarrhœa is, like the preceding, the effect of phlogosis in the mucous membrane of the large intestines. It is the lowest grade of chronicity in the disease under consideration, and one which it is important to mark, as being allied with grades where phlogosis is unequivocal. It corresponds with the chronic gastritis already described.

M. Broussais saw a number of people in Italy attacked with diarrhœa, without any other appreciable cause than the influence of climate, and food of an irritating nature, or of difficult digestion. It was accompanied by no other inconvenience than slight colicky pains preceding each dejection. These people continued to attend to their usual avocations, for some weeks, till the debility and the harrassing frequency of stools, compelled them to give up. As long as the patient continued his usual occupation and regimen, so long the disease continued;—and in such cases it was often protracted for six months. But, by degrees, it exhausted the patients. If they were of an irritable constitution—if they had constringing pains of the abdomen, with a contracted, quick pulse, they usually fell into marasmus. Or if they were of a more relaxed and leucophlegmatic habit, (which is often the case in these people) they became dropsical, and died with, or without, coma, according as effusion did, or did not, take place within the coverings or cavities of the brain.

"In fine," says our author, "when the irritating causes exalt all at once, the action of the gastro-intestinal mucous membranes so far that pain shall suspend their functions and disturb the harmony of their movements—that is to say, when the gastric or intestinal irritation shall become suddenly so powerful as to cause local pain, vomiting, or diarrhœa, and fever—we have *acute inflammation* of the mucous membranes. When, on the other hand, these irritating causes exist for some time, without producing more than such a moderate excitement as shall only suspend the gastric functions for a short time, and feebly call forth the play of the sympathies, without greatly disturbing the general harmonies of the system—then we have *chronic phlogosis* of the same structures." *Tom.* II. *p.* 228.

IV. *Organic Changes.* Every phlogosis of the mucous membrane of the digestive organs, which proves fatal, during its acute stage or state, presents, *post mortem*, the tissue thickened, dense, reddened in different degrees, and sometimes exhibiting the characters of ecchymosis, or even gangrene. The membrane is sometimes found eroded, or, as it were, gnawed in small isolated spots;—and, finally, covered or not, with an exudation of various consistence and character. The change of *colour* from rose red to violet, or even black, does not necessarily prove disorganization of *structure*.

"An attentive observation has convinced me, that patients have often died from the sole effects of *pain*, in early stages, and before the inflamed texture (trame enflammée) was broken, or sensibly altered in its composition. This is the fate of those unfortunates to whom cordials have been given, when overwhelmed with debility resulting from the nervous irritation and pain in such sensible structures. I have often resuscitated, with lemonade, men who were without pulse, delirious, and almost in the agonies of death. And those who have died in this state, frequently shewed nothing more on dissection, than discoloration, without erosion or fœtor, of the mucous membrane."

Here M. Broussais very properly observes that various authentic histories prove how long, the mucous membranes, both of the digestive and respiratory organs, will resist disorganization, when foreign bodies are lodged in contact with them, and keeping up great irritation, and disorder of the whole system. Among a number of examples of this kind, related by M. Dumeril, we shall only select the case of a youth of twelve years of age, who, up to that period, had enjoyed perfect health. At this time he began to evidently emaciate, with frequent and dry cough, evening fever, morning sweats, and other symptoms indicative of consumption, which every day increased in severity. The patient seemed at his last goal, when one day he passed by stool the shell of a nut which he had swallowed twelve or fifteen months before. From that instant the symptoms began to abate, and the patient was soon restored to perfect health. This, and other cases of a similar nature, may lead us to hope for a cure, even in long continued gastritis and enteritis. The above case may give some idea of the endless train of symptoms produced by irritating and morbid secretions passing along the sensible membrane of the bowels from day to day, and keeping up a deranged state of the whole constitution. It elucidates the effects of mild eccoprotic and alterative medicines, steadily persevered in, till the abdominal organs become sound and free in function, and the secretions of a mild and healthy nature.

Our author here supposes that people may object, by observing that patients will often complain of no pain in those parts that are phlogosed—not even when they are a prey to the most terrible anxiety, fever, convulsions, and delirium. But we would answer, that he must be a very bad physiologist as well as pathologist, who would expect that irritation or even breach of substance of an internal organ or tissue shall always exhibit the common feelings or sensations of an organ of sense, as the skin for example. No; these parts have their own organic sensibility, which may produce infinite disorder in the system, before the common sensibility, or feeling of pain be developed. Although pain, therefore, be a strong proof of disorder in an interior part, the absence of it is no proof to the contrary. We must always take various phenomena into consideration.

Those patients who had fallen a little later, after passing from a state of excitement to one of exhaustion, with symptoms of low, putrid fever, especially fœtor of the breath, have often presented, on dissection, the mucous membrane black, easily lacerable, and exhaling a gangrenous odour. This was by no means always the case, even when symptoms led to the expectation of it. Erosions only take place partially, and in the portions most phlogosed. Apparently they are incipient ulcerations.

Fatal *chronic* gastrites have presented to our author disease of the mucous membrane differing somewhat from that produced by dysentery. In Italy he found the same morbid appearances after chronic, as after acute, gastritis—namely, discolouration, thickening, and sometimes erosions. He never saw unequivocal ulceration. The redness was not so deep in chronic as in acute disease—nor did the violet or black parts exale the gangrenous odour. The thickening of the membrane was uniform. In almost all the dissections of chronic cases the digestive tube was found contracted, so as to contain scarce any excrementitious matters, its parietes being almost every where in contact. In very protracted cases the whole of the intestines were found wasted and shrunk, so as to occupy but a very small space indeed. Larry makes the same remark; and Tartra found, in a patient who had been three months ill with gastritis, the intestines so reduced that they could be, as it were, held in his hand. The intestinal calibre did not exceed that of a quill, and in many places was almost completely obliterated. We have seen a few remarkable instances of this kind ourselves; but the peritoneal covering was also implicated in the disease, and the flexures of the intestines all glued together.

M. Broussais remarks that, notwithstanding what has been said, he sometimes found that considerable irritation had continued for two or three months in the mucous membrane, without leaving a single trace of its existence, *post mortem*; death having, apparently, taken place from that excessive debility resulting from the interruption of the digestive process by pain, together with the sympathetic disorder of various other functions of the system. When, however, chronic phlogosis of the mucous membrane was protracted to a much greater length of time, as for some years, which is not unfrequently the case, then there was a different result. Disorganization was evident, and generally consisted in a thickening of the stomach, for several inches in extent, involving the three coats in one confused and morbid structure.

Chronic dysentery always leaves, *post mortem*, a greater or less degree of thickening in the mucous membrane, and generally a number of ulcerations, resembling those of syphilis; the mucous membrane in those places being entirely destroyed, and the muscular coat of the intestine always forming the floor of the ulcer.

A minue examination of such of these ulcers as were in an incipient stage, convinced M. Broussais that they took their origins in the crypts or glandules that furnish the mucous secretions. Around them the membrane was thicker than elsewhere, and of a blackish colour. These ulcerations were most numerous where the fæces are apt to lodge, as the cœcum and lower half of the colon. In some instances, M. Broussais has observed these ulcerations in the lower portion of the ileum, but never elsewhere. M. Broussais concludes, (and there can be little doubt of the justness of his conclusion,) that when feculent matters become fetid and putrid, whether from protracted remora, or imperfect digestion, they prove a source of irritation, and ultimately of phlogosis in that part of the mucous membrane where they happen most to lodge. It is also evident that, when irritation is present in the mucous membrane of the bowels, a greater quantity than usual of mucus is secreted, and this is a characteristic of the class of complaints under consideration. When ulceration of the mucous membrane has taken place, M. Broussais considers the disease as incurable, or nearly so. They do not exist in the stomach or small intestines. There is no sign by which we can ascertain their existence in the large intestines during life.

V. Before entering on M. Broussais' methodus medendi, we shall here introduce an outline of Dr. Abercrombie's opinions on affections of this class of mucous membranes. Viewing the internal coat of the intestinal canal in the double light of a secreting membrane and an absorbing surface, he justly considers that disordered function of secretion must derange the process of absorption, and this latter disorder will have, of course, a great and deleterious effect on the whole constitution, by cutting off the supply of the system.

The morbid conditions of this membrane he refers to acute or chronic inflammation, and their sequelæ, thickening, erosion, and ulceration. As the incipient movements of inflammation here are seldom observed, diarrhœa is generally the first symptom that comes under our cognizance in practice, accompanied by pain in the abdomen, more or less diffused, and usually increased by pressure, but without that acute sensibility attendant on inflammations of the peritonæal covering.

"It differs from peritonæal inflammation also, in being less affected by inspiration and by motion, so that the patient can often bear the erect posture with little inconvenience. The pain, in general, varies very much in degree, leaving long intervals of ease, and then occurring in paroxysms of violent tormina; these are generally followed by discharge from the bowels, but may take place without any discharge following them. In some cases, however, the pain is more permanent, so as more nearly to resemble the pain of enteritis. In general, there is frequency of pulse, with thirst, febrile oppression, and a brownish fur on the tongue; but, in some cases, the pulse is little above the natural standard through the whole course of the disease. There is frequently vomiting, but not urgent, and sometimes a peculiar irritability of the stomach, so that any thing taken into it excites a burning uneasiness, and this is usually followed by an irritation of the bowels, with a feeling as if the article which was swallowed almost immediately passed through them.

" The evacuations from the bowels vary very much both in appearance and frequency. In some cases they are slimy and in small quantity; in others, they are copious; sometimes they are watery and dark coloured; sometimes whitish; frequently yellow and feculent, as in a common diarrhœa; and sometimes articles of food or drink pass through nearly unchanged. They are in some cases extremely frequent, the patient being called to stool every ten or fifteen minutes; in others, the disease may be going on rapidly to a fatal termination, while the bowels are not moved above three or four times in the day. No diagnosis of the disease, therefore, can be founded either on the frequency of the evacuations, or on the appearance of the matters evacuated. In some cases there is tension of the abdomen, but in others this is wanting; and it may appear and disappear several times in the course of the same case." *Ed. Journ. p.* 322.

In this way it may go on for one or two weeks, or extend to five or six—or pass into a chronic state, forming a disease analogous to what is termed lientery, wearing the patient out at the end of several months.

Its fatal terminations, in the acute state, are, according to our author, a peculiar rapid exhaustion—or conversion into peritonitis or enteritis, in which case, the diarrhœa usually ceases a few days before death. Dr. A. lays down little satisfactory as to diagnosis or etiology. We shall here introduce an extract relative to the appearances on dissection, by which it will be seen that Dr. Abercrombie has been anticipated in all essential points, by the French pathologist. The corroboration afforded by Dr. A. however, is satisfactory.

" The appearances, on dissection, vary considerably, according to the period of the disease at which the fatal event takes place. When this happens at an early period, we find the mucous membrane covered with irregular patches of inflammation, which are, in general, sensibly elevated above the level of the sound parts. They vary exceedingly in extent in different cases, in some extending over a great part of the canal; in others, being confined to a very small portion of it, frequently about the lower end of the ileum, or the head of the colon. They vary also in size, consisting, most commonly, of patches one or two inches in diameter, with sound portions interposed betwixt them, above which they are sensibly elevated. In other cases, but I think less frequently, a considerable extent of the canal is of a continued uniform redness.

"The inflamed portions are in some cases covered by a brownish tenacious mucus; in others, by coagulable lymph, and frequently the surface of them is studded with minute vesicles, which, at a more advanced period, seem to pass into very small ulcers. In other cases small round portions of the membrane are observed of a grey colour and soft pultaceous appearance, are found to be easily separated, and to leave ulcers. In the cases which have gone on to a more advanced period, we find ulcers of various extent and appearance. In some examples, they are of the same colour with the surrounding parts, and merely appear as if a portion of the membrane had been dissected out. In other cases they are more decidedly ulcers, covered at the bottom with yellowish sloughs, often with elevated edges, and surrounded by a ring of inflammation, and sometimes penetrating to such a depth as completely to perforate the intestine. These different appearances seem to be different stages of the same disease; for we may sometimes observe one of these penetrating ulcers, surrounded by a larger circle of abrasion, without evident ulceration, and this by another ring of inflammation; this outer inflamed portion being probably covered by the very minute ulcers or small vesicles formerly mentioned. The appearances which I have now described seem to be the most common; but cases occur in which an extensive portion of the mucous membrane is black and gangrenous, and sometimes an extensive portion has been found to be separated, so as to expose the muscular coat, or even the peritonæal. Cases are also described which have recovered, after a portion of the internal coat had been thrown off in this manner, in one continued cylinder of great extent. The external surface of the intestine is sometimes healthy; in other cases there are spots of obscure redness corresponding to the inflamed portions of the mucous membrane. The cases which terminate by peritonæal inflammation or enteritis, have the appearances usual in these affections, and in the cases in which the ulcers penetrate the intestine, effusion of coagulable lymph, lividity, or gangrene, to a small extent, may often be observed on the outer surface, surrounding the perforations." 324.

Dr. A. thinks that acute inflammation of the abdominal mucous membranes is a frequent disease of infants about the age of six or eight months, being with difficulty distinguished from the common bowel complaints of children, resulting from the constitutional irritation of dentition. There is pyrexia, with fretfulness and screaming, bad sleep, frequently vomiting, pressure on the abdomen, in many cases, giving pain. The disease often assumes the character of what is termed "the infantile remittent fever." The bowels are generally, not always, loose, the evacuations being preceded by much restlessness and apparent uneasiness, and being very various in colour, odour, consistence, &c. Sometimes they consist of a reddish brown mucus, sometimes of a pale clay-coloured matter, sometimes of a dark watery fluid, and at others, not varying materially from a healthy state. The disease is usually mistaken for a common diarrhœa, until strong constitutional symptoms, as great febrile oppression, dry crusted tongue, thirst, vomiting, or a rapid exhaustion of the vital powers, excite the attention and anxiety of parent and practitioner. *Dissection*, in these cases, usually shews irregular patches of inflammation in various parts of the inner surface of the intestines, especially the ileum, often covered with minute vesicles or ulcerations. In cases terminating with coma, effusion in the brain is found, often preceded by a remarkable paucity of urine.

Of the *chronic* form of mucous inflammation, in general, Dr. Abercrombie does not say much. He thinks it may be a sequela of the *acute* disease, or an idiopathic affection. When it has continued for some time, we find the patient withered and emaciated, generally with diarrhœa, either constant or alternating with a constipated state of bowels, the appetite being variable and capricious, sometimes good or even voracious. The food often produces uneasiness until it is evacuated imperfectly digested.

If by opiates or astringents the diarrhœa be restrained, the gastric uneasiness is generally much increased, and vomiting is sometimes excited. In other cases, vomiting regularly alternates with diarrhœa, the remedies that relieve the one aggravating the other. The abdominal pain, though generally existing, is various in character, being sometimes in the shape of tormina preceding the evacuations—in others, more permanent and increased by pressure. The matters evacuated are very various—sometimes fluid and feculent—frequently white—and sometimes a mixture of half-digested articles, with recent bile, or brownish mucus, the production of the diseased surface. "In some cases there are discharges of venous blood, which may either appear in the form of coagula, or as a dark pitchy matter, giving a dark colour to the whole of the matter discharged." The *autopsial* observations of our author on the chronic form of the disease differ, in some respects, from those of M. Broussais, and therefore, we shall introduce a quotation from the former in this place.

"The appearances on dissection shew the disease in various stages. In some cases, even after the symptoms have existed for a considerable time, we still find it in the form of irregular patches of a fungous appearance, and a dark red colour, slightly elevated above the healthy parts. In others, we find distinct small ulcers, with round elevated edges, and sometimes more extensive irregular ulceration, with ragged edges. Frequently the coats of the intestines are thickened at the ulcerated parts, sometimes to such a degree as considerably to diminish the area of the intestine. In such cases, the ordinary symptoms of the disease are apt to alternate with attacks of obstinate costiveness, and they are frequently fatal by ileus. In some cases, instead of ulceration, the inner surface of the diseased parts is studded with numerous tubercles, of various sizes, and sometimes an extensive tract of intestine is found covered with smooth cicatrices of ulcers, which have healed. In some of these cases, the symptoms have continued, and gone on to their fatal termination in the usual manner. In others, this appearance is found after the spmptoms have ceased, and the patient has died of some other disease. Cases have also occurred in which the patients died of emaciation, after the symptoms had ceased, apparently from these cicatrices being so extensive as to interfere with the process of absorption." 328.

Dr. Abercrombie justly observes, that there is great reason to believe, that the class of disorders under consideration exists in a degree short of actual inflammation, but sufficient to produce a host of those anomalous affections of the *chylopoietic organs* which, of late, have forced themselves on the attention of various physicians and surgeons. Indeed we are firmly persuaded that, to irritation of the mucous membrane of the stomach and bowels, together with the various sympathetic disorders of other organs, the consequence of this irritation, are owing nine tenths of human afflictions—and that the more this subject is studied, the more power will we have over diseases which have generally proved obstinate or intractable. It was from this conviction that we brought forward the present article, and hope it will not fail to excite to farther investigation.

VI. *Treatment.* M. Broussais thinks he may hazard one fundamental rule, in the treatment, which is without exceptions—namely, that when the internal surface of the alimentary canal is phlogosed—that is, when its sensibility and temperature are exalted—its texture temefied, and its nerves in pain, it cannot bear, with impunity, the application of irritating substances—on the contrary, our prime curative indication is to bring into contact with it, matters the reverse of stimulant. The upper and lower portions of this canal, that is, the stomach and intestines, are somewhat differently acted on by the same substances, and therefore require to be considered separately.

A. *Treatment of Gastritis in general.* M. Broussais thinks that this is simple and easy. This inflammation requires 1mo TIME to subside before aliments are introduced into the stomach—2ndo medicines to facilitate the favourable termination of the disease. The *first* precept or rule is to be most rigidly observed. Meat, the farinaceæ, and fruit ought to be prohibited in gastritis. The best drink is a very dilute solution of gum tragacanth, which is less irritating than gum arabic, the extractive or colouring matter of the latter being somewhat stimulant. While we forbid the use of aliment, or drink of an alimentary nature, so that the stomach may be at rest till the phlogosis is resolved, this resolution may be accelerated by blood-letting, sedative, topical, and other external applications. *General* blood-letting, our author thinks, will seldom be of service—excepting in very acute forms of the disease, when the strength of the pulse, the dyspnœa, or the sympathetic cough, unequivocally demand that measure. Local bleeding, especially by leeches placed over the epigastrium, is of more certain benefit. Yet even this is only an auxiliary, and gives but temporary relief if unaided by internal emollients and sedatives. Of the internal sedatives the vegetable mucilages and acids are by far the best. The *former* must be simple, pure, and free from extractive or aroma, as linseed, althea, quince seeds, gum tragacanth, and others that are perfectly insipid. The decoctions, infusions, or solutions of these substances should be made, if possible, with distilled water, and be very dilute, otherwise they are apt to soon disgust the patient.

After the mucilaginous substances, the vegetable acids stand next in order of utility—but they must not be employed indiscriminately. The acetic acid does more harm than good. The lemon, of all fruits, furnishes the best acid for the drink of a patient labouring under gastric inflammation. After citric comes the pure tartaric acid; but it must be given extremely diluted. Orange juice diluted in water is beneficial, but the patient generally soon tires of it. The gooseberry and raspberry are better. The acid of the mulberry is too penetrating, and if employed, must be very largely diluted. The mineral acids are poisons in these cases. The vegetable acids abovementioned, are only to be employed so as to give a light and agreeable acidulous taste to the water or ptisans used for drink. Saccharine vegetable acids too are to be very sparingly given, as the sugar is slightly stimulant in itself, and, if not digested, runs into the spirituous fermentation. M. Broussais has not employed the carbonic acid, though he thinks it might be useful. Blisters to the region of the stomach do as much harm by their general excitement of the system, as they do good by their local revulsion. He thinks the same observation is applicable to all the other vesicatories or rubefacients. If then, irritation of the skin generally propagates a sympathetic irritation to the internal coat of the stomach, we might expect that such applications to the surface as produced pleasing sensations there, would soothe the gastric affection. Experience, he says, corroborates this. The patient naturally craves for pure cool air, throws his arms out of the clothes, and bares the chest and epigastrium. He has found it extremely useful in this complaint, to keep the surface over the stomach wet with cold, or at most, tepid evaporating lotions, by means of cloths. The application of ice, if the weather be hot, is serviceable, in many cases. "Let not," says our author, "practitioners despise these means last enumerated, as useless or superfluous. I have derived from them the greatest advantage. I have seen patients freed from pain and gastric malaise, almost instantaneously by the application of flannels, wrung out of emollient decoctions, to the epigastric region. The soothing of pain and the increase of perspiration generally resulted from this measure, and were of incalculable benefit." These observations apply to gastric inflammation in general; the different periods and varieties of the disease require corresponding modifications.

And here we solicit the patient and attentive consideration of our brethren. However we may undervalue the inert practice of our French cotemporaries, in acute diseases generally, we are convinced that their treatment is singularly judicious and appropriate in the peculiar class of the phlegmasiæ now under investigation—not only because the phlegmasiæ do not bear so well the depletory treatment of this country, as far as regards bloodletting; but, secondly, because the system of purgation here is particularly miscalculated to allay irritation and inflammation of the inner surface of the stomach and bowels. In fact, we have not been in the habit of sufficiently attending to the great difference of symptoms and treatment, dependant on the structure in which the inflammation is seated. Let us then divest ourselves of all foolish prejudice, and listen to the dictates of reason and experience.

B. *Treatment of Acute Gastritis.* The *prevention* of this disease may be easily gathered from a contemplation of the causes. When gastritis is unequivocally manifested by the symptoms already enumerated, we must not dread debility by cutting off every species of food, and every kind of drink but the bland fluids alluded to, from even drunkards and gluttons. During the first days, then, of acute gastritis, nothing should be permitted internally, but small quantities, at a time, of dilute lemonade, or the other fluids above indicated. This absolute prohibition should continue till the febrile movements and sympathetic nervous disturbance cease. Then, and not till then, should we venture on even the farinaceous decoctions, or the infusions of the saccharine fruits, as apple or pear tea, &c. Nor till this period should veal or chicken broth be allowed. Next, in order of aliment, but not for some days after the farinaceous regimen, panada, bouilli, soup, may be cautiously ventured on. Solid aliments should be abstained from till every symptom is gone, and repeated proofs are offered of the return of the digestive powers. Then they should be of the lightest and tenderest kind, and small in quantity. Here M. Broussais introduces an instructive case in illustration, which we shall insert, but considerably condensed in language.

" *Case* XXVIII. M———, 48 years of age, stout and muscular, had lived, during the last four years, an irregular life, in respect to food and drink, accompanied by some gastric derangements, which were removed from time to time, by gentle evacuations, diluents, and tonics. In October 1807, he committed a great debauch in eating and drinking, and was seized, after retiring to bed, with sudden vomiting and purging. Every kind of drink which he swallowed was instantly rejected, and the stools became black and fetid, but passed without any pain. The pulse was unaffected. This cholera continued four hours. When over, the debility seemed extreme. Antispasmodics, tonics, &c. But soon the pulse rose in force and frequency—the skin became hot—the mouth dry—the tongue coated. In short, he soon presented the characters of low or adynamic fever, and his physician prescribed wine and water. The pulse keeping up, no stronger stimulants than the above were exhibited, and in three or four days the febrile symptoms disappeared. His physician now permitted a little light food and two or three glasses of claret, to recruit his strength, while some rhubarb and manna were given to procure some stools, constipation having succeeded to cholera. Stools were procured, and two days passed thus, in supposed convalescence. But on the third day, the tenth from the attack, high fever, red eyes, loquacious delirium, great restlessness, and surprising change of countenance took place. The idea of typhoid fever now took possession of the physician's mind, and camphorated decoction of cinchona, with stimulant antispasmodics were prescribed. These being ineffectual, recourse was had to sinapisms to the legs. The disease increased, and next day (11th) M. Broussais was called to the patient, who presented the following symptoms:—face distorted—eyes haggard, with the conjunctiva of a deep red colour—countenance that of a person insane, or in the last stage of putrid fever—tongue clean—skin dry and hot—pulse hard, frequent, and rather strong—constipated bowels—all the excretions checked—no gastric or abdominal pain on pressure. When asked how he did, he replied 'very well.' All his expressions were incongruous. He picked the bed-clothes—his muscular powers were extremely depressed—his voice and limbs trembled. The history of the complaint and the phenomena convinced M. Broussais that the commotion of the nervous system was owing to phlogosis of the mucous membrane of the stomach, and prescribed gum tragacanth solution, sweetened with some lemon syrup, to the exclusion of every other kind of ingesta. In the evening the pulse was softer—he had made water thrice abundantly—restlessness was less—and, during the night, some moisture appeared on the skin. *12th day.* The symptoms continued to ameliorate; and on the 13th he had a keen appetite. A little vermicelli. *14th day.* Another vermicelli in the morning. In the course of the day, the fever and delirium rose again. A glyster brought away five stools, the first solid, the others black and fetid. On the *15th day*, he took a little farinaceous aliment, followed by much malaise and debility. On the 16th, in consequence of dyspeptic appearances of the mouth, and great weakness, some light tonics were ventured on. Barley ptisan with syrup of orange peel was given. Quickly there came on great heat of stomach, quick pulse, anxiety, colic, and other disagreeable symptoms. M. Broussais was again summoned, and changed the foregoing drink for lemonade. Great amelioration of symptoms—in a few hours tranquillity was restored; and next day the appetite returned. Two days after this, some rice was imprudently given to the patient, followed by a renewed train of fever, delirium, &c. It was now evident to the most ignorant and prejudiced byestander, that food always reproduced the bad symptoms, and that the stomach was incapable of digesting any thing beyond lemonade, to which the patient was rigidly confined for the next two days. Tranquillity was now secured, and on the 22d day from the commencement of the attack, there only remained debility, which was removed in a reasonable time, by a gradual and careful return to nourishing food."

To M. Broussais, who was long accustomed to see gastric irritation give way, with very little resistance, to diluting drinks, and the most rigorous regimen, it appeared incontestible that the foregoing case would have terminated favourably on the fourth day, had not the febrile movement, succeeding the cholera, been treated as a putrid fever. He attributes the relapse, in the first instance, to the vermicelli, the claret, and the purgative. The subsequent exasperations of the complaint by errors of diet are too remarkable to require notice here. M. Broussais observes, in this place, that delirium is one of the worst symptoms in acute gastritis, as he has seen but few recoveries where it had taken place.

The next case (being the 31st of our author's) which we shall give some account of, was one where acute gastritis simulated malignant fever.

*Case.* Sauriot, 28 years of age, robust and well made, fell ill on the 23d July, 1807, at Udina, during a season of great heat. M. Broussais did not see him till the 28th, the 5th day of the disease. He was then cadaverously pale, and extremely debilitated. He lay on his bed motionless—his eyes closed, and his limbs thrown apart. This state of depression was occasionally interrupted by feeble groans, and contortions of the body, with change of posture when spoken to. He could not pronounce a word. When he opened his eyes, they appeared as though he was moribund. He indicated by signs and gestures that the epigastrium and upper part of the belly were the seat of his sufferings. He refused to take any thing—but when prevailed upon to swallow any liquid, he immediately vomited. Constipation was obstinate. His limbs were cold—the pulse small and slow—the face of a leaden colour—no fœtor of the excretions. Although no history of this case could be traced, yet the season of the year, and the circumstance of many others being ill at the time, convinced M. Broussais that the disease was not putrid fever, but irritation and inflammation of the mucous membrane of the stomach. His determination therefore was soon taken. He prescribed no other medicine than mucilaginous drink, acidulated with citric acid, and "*Lait de Poule,*" for aliment. This plan was continued for six days, during which all the symptoms were gradually ameliorated, but the bowels had not acted. On this account hydromel was substituted, for one day, instead of the mucilaginous drink, and several stools were procured. From this time all went well, and he was discharged to duty in thirty days from his entrance into hospital.

The following case of acute gastritis preceded for a long time, by irritation in the stomach, holds forth important matter for reflection and preventive measures. We shall compress the long original details as much as possible.

*Case.* Mons. P———, 39 years of age, robust and muscular, complained, during the summer heats of 1806, that his appetite was gone—his digestion slow, and his bowels confined. He was grown pallid, and wasted a little. M. Broussais advised him to drink his wine more diluted—to lessen the quantity of his food—and to leave off coffee and liqueurs. The latter part of the advice was not followed. When the cool weather set in, finding his stomach complaint somewhat relieved, he adopted all his former habits. He now became so costive that he rarely had a stool without the assistance of enemata. He felt as though a bar crossed in the line of the transverse arch of the colon, with an obstruction to swallowing from something in the chest.

In this way he continued about five months, when his appetite totally failed for a few days, but by abstinence and diluents returned a little. On the 25th January 1807, having eaten some teal, he felt greatly incommoded. At this time chronic *irritation* seemed to have assumed the more formidable character of inflammation, denoted by the following symptoms:—a most disagreeable sense of weight at the epigastrium, with the transverse bar above alluded to—general malaise—irregular shiverings and flushings which, for two days preceding M. Broussais' attendance, led the byestanders to believe, that Mons. P. had an ague. When our author saw the patient, three days from the eating of the wild fowl, his cheeks were red—countenance desponding—tongue dry, and a little white in the middle—breath somewhat offensive—every thing he swallowed lay like a stone in his stomach—urine very scanty—pulse hard, vibrating, and rather frequent. He was put on lemonade, and ordered to take every half hour a spoonful of a mixture of oil of almonds and lemon syrup. He passed the night much easier than the preceding ones. Next day the symptoms were somewhat mitigated; and fomentations were ordered to the epigastrium, with an oily enema. In the evening the pulse fell—he had no shiverings—passed a good stool, and made abundance of urine.

From this time the recovery was progressive, but slow, and he was not able to return to his regular diet till 34 days from the supervention of the acute form of the disease.

This case, M. Broussais observes, is important, inasmuch as it shews that gastritis, long preceded by irritation in the stomach, may be checked in a few days by diluents and demulcents alone. We shall now introduce a case of chronic gastritis.

C. *Ch. Gastritis.* Danton, 20 years of age, while marching as a conscript to join his corps at Redina, was forced to enter the hospital of Brescia, on account of obstinate and severe pains in his stomach, about the middle of November, 1809. He was not affected with vomiting; but he had lost his appetite, and felt very ill after eating any thing. He remained eleven days in the hospital, without experiencing any relief. He then joined his corps, where he continued in the same sufferings, with such increase of emaciation and debility, as compelled him to enter the hospital of Redina, on the 26th of December, 42 days after the commencement of the disease. M. Broussais found him pallid, dejected, incapable of any exertion, without appetite, and costive—in short, in an incipient state of marasmus. He complained of obtuse and deep-seated pain in the epigastrium, accompanied with constant malaise. The region of the stomach was somewhat full, and when pressed on occasioned pain. The pulse was small and hard, and rather frequent—skin dry to the touch, and hot. Believing all these symptoms to be dependant on gastric irritation, our author considered that only mucilaginous emollient aliment and medicine were necessary to deprive the gastric membrane of its excess of sensibility. For two days, therefore, the patient was confined to mucilaginous and acidulated drink and weak broth—then some bouilli for diet. He did well. During convalescence some antispasmodic and anodyne medicine was given him, for the purpose of procuring better sleep. A relapse immediately occurred, and he was forced to adopt the same rigid system as before. The convalescence was tedious, but ultimately health was completely restored.

Here M. Broussais observes that, in the treatment of chronic gastritis, we are not so much to regard the length of time which the disease has existed, as the degree of debility or marasmus to which it has reduced the patient. So long as the muscles are not greatly extenuated, marasmus is not to be considered as established; and although the patient appears excessively debilitated, we must not be in haste to exhibit tonic and strengthening medicines or food. Strength is not annihilated, but temporarily subdued by the gastric irritation and pain. When to the absence of extenuation is added a knowledge that the disease was slight at first, and maintained by improper stimulants, we have every reason to hope for recovery by rigid abstinence and aqueous emollients. It is surprising how rapid the change, sometimes in such cases, from sickness to health, by the simple means abovementioned. "*La promptitude de l'amelioration est plutôt due à l'absence de toute irritation, qu'à une vertu specifique des medicamens.*" We consider an abridgement of the following case not unworthy of record here.

*Case of Chronic gastritis.* Meurat, a gunner, 32 years of age, was treated in one of the hospitals of the Frioul, in May, 1807, for an intermittent fever, accompanied with vomiting during the paroxysms. He was given emetics, and afterwards the bark, which suppressed the fever, and he was soon discharged to duty apparently well, except that his stomach was rather irritable. On the 15th day after his discharge, he was suddenly seized with vomiting of his food, on account of which he drank a little more wine than usual, to strengthen his stomach. In this state of gastric irritation he continued fifty days. But the vomiting becoming more violent, and accompanied with very severe pains in the epigastric region, lassitude, debility, and general malaise, he was sent to the hospital of Udina, on the 14th of July, 1807, on the 50th day of the vomiting, and about two months and a half after the cure of his ague. His eye was now sunk—his complexion that of lead, with a yellowish tinge—adipose membrane entirely absorbed—muscles not extremely extenuated—the whole surface cold as a corpse—pulse scarcely perceptible—inability to sit up—constant jactitation—deep and plaintive sighings—throws the clothes off his chest, and his arms out of bed—nearly incapable of speaking—quite delirious. He vomited every thing that was given to him, and continued to strain violently after the stomach was empty. The epigastrium being pressed evidently increased his sufferings. He was every instant at the close-stool, but passed only mucus and blood. He was ordered to have nothing but solution of gum arabic, or sweetened linseed tea, and during the first three days, he had nothing in the shape of aliment, except a little "*lait de poule*."* On the fifth day from his entrance into the hospital, the vomiting ceased, and on that day he had but two stools. The pulse was now developed, but hard and quick—the skin warm and moist—the delirium had disappeared two days after his arrival—craved for some food—the epigastrium still very tender to the touch. Very weak broth in the morning—lait de poule at night. The epigastrium gradually lost its tenderness, and in two or three days the febrile symptoms subsided. From that time the patient began to gain flesh and strength, and in three weeks from his arrival in the hospital he was discharged to duty cured.

The above and many similar cases may convince us that the subduction of stimuli alone will go far to cure inflammatory irritation of the mucous membrane of the stomach; and it may serve to restrain a little the "*medicina perturbatrix*," or, at all events, the *poly-pharmacy* of British practice, which too often crams the stomach with medicines, without discriminating minutely between affections of the inner and outer coverings of this important organ and its appendages, though such a discrimination is of infinite importance in the treatment of the case. We seriously caution our brethren against resolutely shutting their eyes against this simple and apparently inefficacious practice of continental physicians, *in the class of complaints now under consideration*, for we are far from approving the practice generally in acute diseases of other structures in the body.

D. *Chronic Latent Gastritis.* In by far the greater num-

* The yolk of an egg beat up with a little sugar and rose water.

ber of cases, gastric irritation does not, at the beginning, create that degree of disturbance in the animal economy which may clearly indicate the nature of the disease, and therefore it passes, among the generality of observers, for what are vaguely termed *bilious* and stomach complaints. How are we, in such cases, to discover the true state of affairs? An enquiry into causes, modes of life, climate, occupation, &c. will often furnish us with strong grounds for presumption, and an attentive observation of the progress of the complaint will do the rest. Gastritis so slight as not to be recognized by the general symptoms, enumerated in their proper place, will not, M. Broussais thinks, be materially injured by the administration of an emetic. A temporary mitigation, indeed, of the symptoms generally ensues. The relief, however, is only temporary; but the subsequent recurrence of the symptoms is a valuable diagnostic. Whenever therefore we find fever, pain, and anorexia developed, after such a process, there is no longer any doubt upon the subject, and then rigid abstinence, with the bland diluents, before described, are to be immediately insisted on.

In the summer of 1806, M. Broussais had a great number of these cases under his care. He began, in all cases, with the plan of abstinence and diluents, and the majority of the cases speedily got well. Where anorexia, bitter taste in the mouth, nausea, dryness of the skin, eructations and flatulence continued, then the patients were vomited, and the appetite immediately returned. The bowels, however, in these cases, required purgatives to clear away the saburra remaining in them. In some cases, indeed, febrile movements continued after the operation just mentioned, and in these a kind of bilious fever ran a course of four, five, or six days. Even here there was nothing else required than aperients and diluents—no bitters, no tonics were given. There was another class of patients that came under M. Broussais' care, with the following obscure symptoms:—paleness of the countenance, without any yellow tinge—some trifling appetite for breakfast, but not afterwards—after eating any thing in the evening, a sense of plenitude, as though they had over eaten—no pain, but a sense of undefined weakness, with tottering of the limbs, habitual constipation, and slow pulse. When to these patients were given bark, bitters, or other kinds of tonics, then the pulse was raised, febrile heat and other pyrexial symptoms excited, and in a short time, unequivocal gastritis was developed. M. Broussais therefore treated these cases in the same manner as chronic phlogosis of the mucous membrane of the stomach, and firmly believes that, by so doing, he saved a great number of lives.

"Those physicians," says our author, "who have profited by the lessons of M. Pinel, well know that this experienced practitioner never lost an opportunity of recommending the most mild vegetable and unirritating regimen to those hypochrondrical, melancholic, vapourish, and (supposed) obstructed patients, who, after having exhausted the whole catalogue of deobstruents, stomachics, purgatives, &c. &c. applied to him to put an end to their sufferings. Numbers have I seen cured by him, after abandoning their drugs, to live only on panada, milk, eggs, and fruit. But it is not easy to persuade the upper classes of society, especially after they have been habituated to spirituous liquors, to betake themselves to food and drink which they consider as insipid, and which, for a short time, appear to be productive of debility. Yet they have but to persevere, for some time, and they will find this regimen not only very pleasant, but a most effectual mode of restoring them to health." *Tom.* II. *p.* 341.

We shall pass over M. Broussais' observations on the treatment of complications of gastritis with intermittent fever, in order to come to the *methodus medendi* in phlogosis of the mucous membrane of the *intestines.*

VII. The phlogosis in question but rarely affects the lining membrane of the small intestines. When it happens, in particular cases, to pass the valve of the colon, and ascend towards the stomach, it generally proves fatal. M. Broussais therefore only treats, in this place, of phlogosis of the mucous membrane of the colon—and first of the treatment in acute colonitis, as the grand preservative from the chronic state. Although M. Broussais considers dysentery to be essentially inflammation of the inner coat of the intestines, yet he believes that, as the same miasmal causes (animal or vegetable) which produce typhus and other contagious fevers, produce dysentery, so the phlegmasiæ, in these cases, are so modified by the causes, and rendered so intense, as often to end in gangrene before art can interfere to prevent such a fatal termination. It is perfectly evident that the wide-wasting dysenteries of our colonial possessions would almost invariably destroy their victims under the French system of treatment, and therefore M. Broussais very wisely declines entering on the subject at all. He confines his observations to those simple phlogosed states of the lining membrane of the large intestines particularly, which, attacking a man in perfect health, do not acquire violence, excepting by maltreatment. From the moment that acute colonitis is declared, the patient must not only abstain from all stimulating drink, but from every kind of alimentary substance capable of leaving any fœcal residue in the bowels. Notwithstanding the atrocity of the pain, and the sense of debility with which the patient is overwhelmed in the intervals, we must not depart from the foregoing principle, so long as emaciation is absent.

The medicines which M. Broussais has found most proper to diminish the intestinal irritation, are the mucilaginous and farinaceous substances. In high states of irritation, he thinks that gum water *only* should be allowed—rice water is then too stimulant, because it requires some digestive effort. Even the simplest mucilaginous drinks are not to be indulged in too freely lest by their very bulk they should act as extraneous bodies. This plan must be persevered in, as long as the stools are frequent, and the tenesmus violent. Mean while, fomentations and cataplasms over the whole abdomen will be very useful. All kinds of enemata he considers as worse than useless, excepting in the very onset of the disease, where fæces may be keeping up additional irritation. In such cases their expulsion should be attempted, first by oily or mucilaginous lavements, and, if that be not sufficient, by manna or other mild purgatives.

> "Au reste, tous ces moyens évacuans ne sont indiqués qu'autant que les excrémens sont opiniâtrement retenus. Le plus souvent ils sont inutiles, parce que le premier effet de l'irritation dyssentérique est de débarrasser l'intestin de toutes les matières qui y séjournaient. Cela étant fait par la nature, il suffit à l'art de ne pas fournir de nouveaux excrémens." *Broussais*, 356.

We agree with M. Broussais, that it is the part of the physician to prevent, as far as possible, the formation of excrementitious matters in the intestines by means of rigid abstinence; yet such is the determination of fluids to the mucous membrane of the bowels, that the most severe regimen will not prevent a great generation of morbid and acrid secretions there. On this account, oily laxatives are frequently necessary in the course of dysentery, though we condemn, as much as he can do, the daily administration of purgatives, in a disease, the principal feature of which is high irritation, if not inflammation of the lining membrane of the intestines. M. Broussais calls in the assistance of the warm bath, rubifacients, blisters, and frictions, when the dysentery appears to be the result of a violent crisis, or a metastasis of inflammation or irritation from some other tissue. "Opium, dans tous ces cas, et fort utile; mais tous ces moyens sont, pour ainsi dire impuissans, sans le concours du régime que nous avons recommandé." Our author affirms, that this strict abstinence succeeded, in almost all cases, however violent, provided the patient came early under his care. We cannot say nay to this practice, never having given it a fair trial; but we have no hesitation in believing that it would, at all events, be an excellent basis, or ground-work for the treatment of dysentery in this country, and even in the colonies. We should not certainly trust to it alone, knowing as we do, the indomitable nature of the disease when suffered to advance towards alteration of structure in the intestinal lining membrane. Broussais seems to despise all means of turning the tide of the circulation towards the surface of the body by internal medicines, and also to dread opium, excepting in some particular cases. Yet we can assure him, and it is now universally known in this country to all who have had any experience in the disease, that a combination of sudorifics and anodynes, with a proper proportion of a mercurial preparation, is by far the most powerful means we possess in allaying the intestinal irritation, and determining to the skin. In this country very severe bowel complaints may be safely and effectually treated with cretaceous mixture and tincture of opium, after each liquid motion, while five or six grains of the blue pill with a quarter of a grain of opium, and three or four grains of antimonial powder, are taken twice, thrice, or oftener in the 24 hours. During this time the patient should be confined to bed, and the Broussaian regimen strictly adopted.

*Treatment of Chronic Enteritis.* A diarrhœa continuing after the 30th day must depend, M. Broussais thinks, on *organic* derangement of the mucous membrane of the colon. In general, however, it is kept up by improper regimen or medicines only. In all cases it is a chronic phlogosis, the treatment of which, he conceives, may be fixed by invariable rules. These rules may be easily anticipated by what has already been said. The food must be such as leaves the least feculence to pass along the intestines; and, of course, the quantity should be no more than the stomach and duodenum can digest, otherwise the remains, when mixed with bile and mucus, will undergo fermentative and other decompositions in the track of the intestines, which, with the gases evolved, create much irritation there. On this account, gentle tonics are serviceable, by strengthening the tone of the stomach; but care must be taken that their action does not go beyond the stomach, otherwise they are likely to aggravate the complaint. The remains of animal food, our author thinks, are much more injurious than those of vegetable; but of vegetables there must be much selection—the farinaceæ alone are admissible in chronic bowel complaints. Barley, wheat, and rice, are preferable to all others. Bread produces too much feculence. Fine flour boiled with milk, forms (as we have experienced) a most excellent diet for chronic dysenterics. Broussais cured great numbers of patients in the military hospitals by this diet.

"Dans la pratique civile, il y a, pour entretenir la nutrition d'un diarrhéique, sans lui laisser beaucoup d'excrémens, mille ressources dont on est privé par les régemens des hôpitaux militaires. On trouvera dans les semoules, les gruaux, les pâtes ou vermicelles, pourvu qu'ils soient très-fins, des moyens de varier agréablement la nourriture, en combinant ces diverses substances avec le lait, la crême, les œufs, le sucre, selon le goût du malade et le degré de sa faculté digestive. 262.

When the digestion is pretty good, meat broths may be cautiously allowed. Whenever they render the stools more frequent, they are to be discontinued, at least for a time. Sweet and ripe fruits, rejecting the seeds and rinds, may be sometimes allowed as a variety.

In chronic phlogosis of the mucous membrane of the colon —in other words, chronic bowel-complaints, our author reduces the list of medicinals to two classes—stomachics and anodynes. When the general erethism attending the commencement of the complaint is completely subsided, but some local pain remains, M. Broussais prescribes solutions of gum tragacanth rendered gently anodyne by Sydenham's laudanum, varying the dose from 12 to 50 or 60 drops of the tincture. These medicines will not be serviceable unless combined with the rigid regimen before alluded to. As the dysenteric symptoms subside, gentle tonics, and gradually increasing aliment are to be allowed. Astringents, at all times, M. Broussais thinks, augment the phlogosis by checking the alvine evacuations. A little wine, or an infusion of cinchona or canella alba extremely weak, is sufficient to solicit the stomach to a resumption of its functions towards the close of the disease. Ipecacuan, as a vomit, our author has not found useful, excepting at the very beginning, when the stomach happens to be loaded. It does not possess, he thinks, any anti-dysenteric properties. The action of vomiting he does not believe to possess any power in checking the increased peristaltic motion of the intestines. When phlogosis of the mucous membrane of the bowels is the cause of the purging, vomiting cannot arrest it: —if the diarrhœa depend on irritating matters in the primæ viæ, then it does harm by checking the operation of Nature in removing them. In short, it is not the mere *symptom* of purging that we are to combat, but the cause of the phenomenon.

From a number of experiments made, with tincture of opium on eruptions of the skin, M. Broussais comes to the conclusion that its first effect is highly stimulant, quickly followed by a sedative operation. He endeavours to explain its beneficial effects in dysenteric complaints on this principle—namely, that, though it may give a momentary increase of excitement to the stomach and upper intestines, a torpor is subsequently induced, which allays the spasmodic contortions of the colon for a certain time. The precautions which he thinks are necessary in administering this remedy in the class of diseases under review, are $1^{mo}$ never to exhibit opium when a *general* inflammatory diathesis prevails. $2^{ndo}$ to forbid the use of this remedy when the stomach is phlogosed. $3^{tio.}$ to abstain from laudanum until the bowels are cleared of all stercoraceous or bilious secretions, either by the spontaneous operations of Nature, or by the effects of aperients administered for that purpose. $4^{to}$ to exhibit this anodyne at first in small doses, and in a demulcent vehicle, when the local erethism is considerable; gradually augmenting the dose till the effect of procuring some sleep is obtained, moderating, if necessary, the stupifying effects of the opium by vegetable acids.* We shall give M. Broussais' rationale of the operation of opium in dysentery in his own words.

"L'excitation passagère que sa première impression détermine, est fort peu ressentie par le colon phlogosé: c'est l'estomac principalement qui doit la supporter; il ne faut pas qu'elle aille jusqu'à augmenter sensiblement l'activité de l'appareil circulatoire. Au contraire, la stupeur toujours plus prolongée qui succède à cette stimulation, est partagée par toutes les ramifications nerveuses, et surtout par celles qui se distribuent dans les fibres musculaires et dans les papilles de la partie souffrante." 371.

We may here observe, that we believe the above observations of M. Broussais to be correct, as relating to the exhibition of opium *alone* in dysenteric affections. Our continental neighbours have such a dread of poly-pharmacy, that they deprive themselves of many valuable combinations used in this country. They have no idea that a combination of opium and antimony or ipecacuan, or of both of these with mercury, can have any better effect than these medicines separately administered. In this country we know better. Ample experience has demonstrated the safety with which

* "Care must be taken that in administering vegetable acids the opium be no longer in the stomach, since, according to the experiments of Orfila, a mixture of acids and narcotics irritates and even inflames the gastric mucous membrane."

opium may be given, in the above and other formulæ, during even acute inflammation of various organs; and in no class of complaints is this combination so powerful and beneficial, as in that which forms the subject of this essay. We trust, however, that the investigation which we have entered into here, will have no inconsiderable effect in restraining the practice of *purging* in dysenteric affections of the bowels in this country, where it has reigned since the days of Cullen—the words, "*retentis plerumque fecibus alvinis,*" appearing to have radiated from alma mater the dangerous principle of *purgation,* as almost the sole remedy. The subject of the present article being one of the highest importance, we will be pardoned, we hope, for detaining our readers some time longer, while offering for their attentive consideration a few necrological histories from the eminent pathologist whose work is under review; which histories we shall abbreviate as much as possible, while we are select in our choice of those which are most likely to leave a salutary impression on the mind.

*Case of Acute Gastritis simulating Catarrh and low continued Fever.* "M. Beau, sub-assistant surgeon of the 18th regiment, 24 years of age, had had several attacks of severe catarrhal affection, and also of hæmoptysis. He was very studious, and had undergone great fatigues at an hospital in Gorizia. While there, he breakfasted for a fortnight on bread soaked in sugared wine, being before accustomed to coffee. This regimen he found heated him much, and rendered him more than usually irritable. He sent for M. Broussais on the 7th March at Udina, who found him complaining of unpleasant heat in the stomach, want of appetite, considerable fever, pulse full, hard, occasionally intermittent, intense heat, but little thirst, pain in the chest, with sense of constriction, great anxiety and restlessness, inclination to cough, but fear of throwing the respiratory muscles into action, on account of the pain. *The pulmonary irritation and force of the pulse indicated venesection, but the intermissions of the pulse, and the patient's having been in an hospital where typhus prevailed, prevented M. Broussais opening a vein!* A decoction of figs therefore, and a blister to the sternum were prescribed, but the blister was not acceded to. Next day all the symptoms were aggravated, and the young man himself loudly demanded to be bled. Broussais, instead of complying, ordered eight leeches to the epigastrium. The young man applied sixteen. The leeches bled profusely, and the flow of blood could hardly be stopped. Next day there was great faintness, with some delirium; but the pulse was down, the thoracic pain gone, and scarcely any cough. M. Broussais ordered decoction of bark, with a little wine, and gummy emulsion. These were vomited as soon as swallowed—(and fortunately too we think)—the same followed the exhibition of aromatic and antispasmodic juleps. Gum water with lemon juice succeeded better. In two days the pulse rose again, with anxiety, and efforts to cough—gum water acidulated continued. It is needless to say, that the patient got worse and worse—a consultation was called, but the new physician saw putrid fever instead of gastric inflammation in the case; stimulants were lavished on the patient—"les stimulans de toute espece furent prodigiés." "The unhappy youth had no longer strength to vomit them up, but his cruel sufferings augmented in proportion to the quantity of medicine he swallowed," and he died on the 16th day of his illness.

"*Dissection.* Pia mater, particularly over the left hemisphere, strongly injected—cerebral substance firm and red—ventricles a little dilated with serum. In the chest every thing sound. In the abdomen, the stomach was found contracted to the size of a small intestine—of firm consistence—the mucous membrane thickened, and throughout its whole extent of a livid red colour, in some points black. All the intestines contracted, and their mucous lining of a shining red colour."

This case happened when M. Broussais was opening his eyes to the disastrous effects of Brunonian practice in fevers, and when he was beginning to discover local inflammation, especially of the mucous membrane of the primæ viæ, as the basis of many reputed idiopathic fevers. The practice therefore detailed above, may be not only accounted for, but, in some degree excused. Our author has no hesitation in believing that he might have saved the patient, if he had confined him rigidly to mucilaginous drink from the time he first saw him. It was this case that first led him to question the power of bloodletting in *completely* subduing muco-gastric inflammation. Further experience confirmed him in the belief that bleeding has but a very subordinate control over phlogosis seated in the hollow and thin viscera, especially in their inner surfaces. It is over inflammation of the solid and parenchymatous viscera, rich in sanguineous capillaries, and the membranes that cover them, that bleeding exerts a masterly control. The cough in this and in numerous others under his care, was purely sympathetic, arising from irritation of the extremities of the par vagum in the stomach. In respect to the brain, the central organ of sensation, how can we expect that this part can escape in diseases accompanied with much pain. "Tel organe qui n'éprouvait d'abord qu'un trouble sympathique, peut s'affectuer organiquement par le seul effet de la douleur." This we are sure is sound pathology.

*Acute Gastritis imitating low Fever.* Venter, a young man of 22 years, presented himself to M. Broussais on the 1st July, 1807, with symptoms of stomach affection, viz. anorexia, slight nausea, some prostration of strength. These symptoms had been of six days standing. Suspecting gastric susceptibility bordering on phlogosis, M. Broussais only prescribed demulcent and acidulated drinks. He grew quickly better, which induced the Doctor to grant him some victuals, especially as it seemed hard to refuse them to a man, who all day walked about the wards and corridors of the hospital. After spending five or six days in this ambiguous state, the young man began to complain that he passed his nights badly—that he had cold chills, and that his mind was confused. M. Broussais thought he could not run much risk, in ordering for these symptoms, some doses of cinchona and a little wine. Two days of this treatment produced no amelioration, and M. Broussais paid the patient a visit at night. He found the young man with hot skin, contracted visage, accelerated pulse, and restlessness. He now became convinced that he had to deal with obscure gastritis, tending to the acute form. The most rigid regimen was, therefore, instantly adopted, namely, mucilaginous acidulated drinks. But he was not relieved—there was delirium at night—trembling efforts to get out of bed—grinding of the teeth. He expired the next evening.

*Dissection.* No appreciable lesion in the head or chest. The mucous membrane of the stomach thickened, red, and, in some places, even black. The inner surface of the intestines presented the same appearance. From the stomach to the anus nothing was to be seen in the alimentary canal but a white firm exudation, very difficult to detach from the inflamed surfaces of the guts.

The following case is still more insidious than the one just related, inasmuch as it masked the greatest malignity under a benign and perfidious aspect, uniting the rapid march of *acute*, with the symptoms of *chronic*, gastritis.

*Case of Acute Apyrectic Gastritis.* Rapion, 25 years of age, robust and well made, had, for several weeks past, lost his appetite, and felt some nausea at the stomach. He took (of his own accord) an emetic, which exasperated the symptoms. Entered the hospital, on the 5th June, 1806, five days after taking the emetic. He had now anorexia, constant nausea, head-ache, slight febrile movement, and relaxation of the bowels. On a more minute examination, M. Broussais found that Rapion vomited his food, and that he had a constant pain in his stomach, extending to the whole of the abdomen, with a sense of constriction, small, frequent pulse, skin rather cold than warm, tongue natural, strength undiminished. M. Broussais suspected gastritis, and prescribed mucilaginous acidulated drinks, with fomentations to the epigastrium. During the next four days there was no alteration in the symptoms. On the 5th day M. Broussais found the patient stretched on his bed, with his clothes on, for his sufferings and the diarrhœa did not permit him to lie in bed undressed. He appeared somewhat delirious; complained of being very ill;—yet his strength was not greatly prostrated. A few hours afterwards he was seized with convulsions and horrible anxiety. He fell into a state of syncope, and expired.

*Dissection*. Nothing particular in the chest. In the abdomen the whole intestinal canal was contracted—the mucous membrane of a deep red colour, and thickened from the cardiac orifice of the stomach to the anus, but without ulceration.

The following case of *chronic* gastritis will exemplify the effects of good and of bad regimen.

Papillon, 22 years of age, entered the hospital of Udina on the 18th July 1805, complaining of distaste for all kinds of victuals, and constant inclination to vomit, with diarrhœa. He reported that he had been ill but sixteen days, yet he was considerably emaciated. His tongue was moist and clean, his pulse nowise febrile. M. Broussais pronounced the disease to be chronic gastritis, and put the patient on acidulated mucilaginous drinks, with a very little bouilli for food. At the end of three or four days the nausea and diarrhœa were considerably mitigated—the appetite had returned a little, and in four days more was very considerable; the diarrhœa being reduced to two or three motions in the 24 hours, and those passed without pain. Still M. Broussais considered it dangerous to allow the patient more solid or abundant food than a little soup, or rice with a small quantity of bouilli. All at once, however, the patient was found complaining of pain in the stomach, nausea, vomiting, and great increase of the diarrhœa, with tenesmus. M. Broussais caused his bed to be searched, and it was discovered that the patient had gorged himself with cold meat and bread. From this relapse till the patient's death, which took place twelve days afterwards, he vomited every thing which was taken into the stomach. The diarrhœa amounted to severe dysentery, and the march of the marasmus was surprising. All this time the circulating system shewed no evidence of febrile movement.

Dissection. Body greatly emaciated—vessels of the head engorged, and the cerebral substance red and firm—thoracic viscera sound—stomach so contracted as to leave no cavity—intestines greatly contracted also—mucous membrane thickened, dry, and of a deep red colour, resembling linen steeped in port wine—the mesenteric capillary vessels much injected, while their trunks contained hardly any blood. The serous or peritonæal covering of the intestines unaffected. The body, when opened, exhaled a remarkable ammoniacal odour.

We find that our limits will not permit us to introduce more than another case.

*Chronic Muco-Enteritis, propagated to the Stomach.* A drummer in the 9th regiment of the line, 24 years of age, slender, but active, became affected with an intermittent fever for two months, in Germany, without using any remedy. He was then sent to an hospital, two months after which he came under M. Broussais at Udina. He was now emaciated in the second degree—had from five to six stools *per diem*, with colic and much uneasiness. The pulse was nowise febrile. He was placed on rice water, mucilaginous acidulated drinks, and farinaceous aliment. In a few days the diarrhœa was reduced to two stools *per diem*, without pain—the appetite returned—the complexion cleared, and the patient was in full march towards convalescence. M. Broussais now ventured to gradually increase the food until he came to $\frac{2}{3}$ds allowance or more. All at once the diarrhœa and colic returned, and in three or four days, he lost all the flesh he had gained in thirty. He grew rapidly enfeebled. M. Broussais now learned that the impatient patient, tormented with his appetite, had purchased provisions from his comrades frequently. He was now put back to the original regimen; but too late! In ten days he was reduced to a complete state of marasmus, although the bowel complaint was moderate, not exceeding two or three stools daily. Vomiting now came on, with entire loss of appetite, great anxiety, frequency of pulse, and heat of skin. It was now evident that the phlogosis had reached the stomach. Yet he lived twenty days longer, when he died a perfect skeleton. On dissection, the whole alimentary canal was found so contracted, as to have all its internal surfaces almost in contact.

In the stomach, this membrane was red, thickened, and covered with a yellowish exudation about the pylorus. Throughout the whole of the intestinal canal, the mucous membrane was dry, and of a logwood colour, with scarcely any fecal contents.

Before closing this paper we shall introduce an extract from Dr. Abercrombie, shewing that gentleman's sentiments respecting the treatment of affections of the mucous membrane of the alimentary canal.

"The active form of the disease, especially in its early stages, is to be considered as an inflammatory affection of the most dangerous kind, and requiring to be treated with activity by the usual remedies —especially bloodletting. The first urgency of the inflammation being thus subdued, if the pulse continue frequent, digitalis is given with much advantage, or Dover's powder, in repeated doses; and, after the necessary bleeding, moderate opiates may be given, with mucilaginous articles and absorbents, or opiate glysters. The effect of purgatives is extremely ambiguous. In the more severe cases, they evidently aggravate the symptoms. There may be cases in which it is expedient to evacuate the bowels, as when the discharges are scanty and slimy, with retention of natural fæces, but the practice requires caution; and in the more common form of the disease, with copious discharges, they appear to be injurious. Though the evacuations in such cases may be of an unnatural appearance, it is to be remembered, that this is the result of morbid secretions, not to be corrected by purgatives, but by curing the disease on which they depend. When the disease appears to be seated in the lower part of the great intestine, bleeding from the hæmorrhoidal vessels might probably be useful. When the tormina are severe, with tension of the abdomen, the tobacco injection might probably be employed with benefit. Great attention should be paid to the ingesta; to keep them in as small quantity as possible, and of the mildest quality.

"It is in infants that the disease most frequently occurs to us; and there is some difficulty in determining what is the best treatment. This results from the difficulty of distinguishing the disease, so that, when a case terminates favourably, we cannot say, with certainty, that it really was an example of this dangerous affection. In some cases, in which there is no vomiting, a gentle emetic seems to be useful in the early stages; afterwards, Dover's powder, combined with chalk, opiate glysters, opiate frictions, opiate plaster, and tepid bath. In some cases, the free use of digitalis seems to be extremely useful, and blistering on the abdomen. It is worthy of consideration, whether topical bleeding would be admissible in the early stages, when the disease exhibits much activity. In the advanced stages, when there is a tendency to sinking, wine is to be given very freely; when there are threatenings of coma, blistering on the neck should be employed; from both these conditions, infants often make most unexpected recoveries. When the case is accompanied, as it often is, by a peculiar and most ungovernable vomiting, blistering on the epigastrium seems to be the most effectual remedy; and considerable benefit, on settling the stomach, is often obtained from the vegetable bitters, as the powder of colombo root, in doses of a few grains, repeated at short intervals. In the protracted bowel complaints of infants, in which there was reason to apprehend this affection in a chronic form, I have found nothing so useful as lime water. The teeth are to be attended to, and the gums cut, when they appear to be giving irritation.

"In the chronic form of the disease, what we have to contend with is either the chronic fungous inflammation, or ulceration. The treatment is extremely precarious, and very few of the cases end favourably. The remedies to be kept in view, and which appear in some cases to be useful, are chiefly the following: Lime-water, the vegetable bitters, and astringents, especially the cortex cuspariæ and logwood; preparations of iron, as the tincture of the muriat, in large doses; small quantities of mercury, with opium; the resins, as turpentine and bals. copaivæ, combined with opium; sulphur, with opium; repeated blistering on the abdomen; bandaging with a broad flannel roller; the tepid salt water bath." *Ed. Journal, p.* 347.

In respect to Dr. Grimaud's Dissertation on Inflammation of the *Follicles* of the Mucous Membrane of the Intestines, as distinguished from common Inflammation of the Membrane itself, we have only to say that we do not believe such a distinction to exist in nature—and, if it did, we are quite sure that it would never be recognized in the living subject, at least, by Dr. Grimaud's diagnostics. We shall not, therefore, waste time in entering into any analysis of the paper. Those who are more curious, may refer to it in the Journal Complimentaire, for December 1820.

It is somewhat curious that Broussais' work should never have been translated into the English language, while several very indifferent publications have been honoured with that distinction. We confess that a literal translation would subject the English reader to a great loss of time, in consequence of endless repetitions, and redundancy of cases, with which the work is swelled out to two volumes. We are disposed to think, that we have rendered a translation, in a great degree, unnecessary, by analysing the different parts of it in this article, and in the eclectic review on peritonæal inflammation. To most of our readers, the original work is virtually unknown, and to *them* we think we have offered something valuable. To those who were previously acquainted with M. Broussais' work, we hope this concentrated analysis of its most interesting features, will not be unacceptable, as a refresher of their memory, when, probably, they would not again go through the original. At all events, we have endeavoured to be useful to our brethren on the present occasion, and can only hope that our labour has not been thrown away.

