# Errata

**Published:** 1822-03-01

**Authors:** 


### ERRATA.

No. 5, page 1, line 9 from bot. *for* complimentaire *read* complementaire

